# Pan-cancer analysis of differential DNA methylation patterns

**DOI:** 10.1186/s12920-020-00780-3

**Published:** 2020-10-22

**Authors:** Mai Shi, Stephen Kwok-Wing Tsui, Hao Wu, Yingying Wei

**Affiliations:** 1 School of Biomedical Sciences, The Chinese University of Hong Kong, Shatin, New Territories, Hong Kong, SAR China; 2 Hong Kong Bioinformatics Centre, The Chinese University of Hong Kong, Shatin, New Territories, Hong Kong, SAR China; 3 Department of Biostatistics and Bioinformatics, Rollins School of Public Health, Emory University, 1518 Clifton Road, Atlanta, Georgia, 30322 USA; 4 Department of Statistics, The Chinese University of Hong Kong, Shatin, New Territories, Hong Kong, SAR China

**Keywords:** DNA methylation, Differential methylation, Pan-cancer, Cancer epigenomics

## Abstract

**Background:**

DNA methylation is a key epigenetic regulator contributing to cancer development. To understand the role of DNA methylation in tumorigenesis, it is important to investigate and compare differential methylation (DM) patterns between normal and case samples across different cancer types. However, current pan-cancer analyses call DM separately for each cancer, which suffers from lower statistical power and fails to provide a comprehensive view for patterns across cancers.

**Methods:**

In this work, we propose a rigorous statistical model, PanDM, to jointly characterize DM patterns across diverse cancer types. PanDM uses the hidden correlations in the combined dataset to improve statistical power through joint modeling. PanDM takes summary statistics from separate analyses as input and performs methylation site clustering, differential methylation detection, and pan-cancer pattern discovery. We demonstrate the favorable performance of PanDM using simulation data. We apply our model to 12 cancer methylome data collected from The Cancer Genome Atlas (TCGA) project. We further conduct ontology- and pathway-enrichment analyses to gain new biological insights into the pan-cancer DM patterns learned by PanDM.

**Results:**

PanDM outperforms two types of separate analyses in the power of DM calling in the simulation study. Application of PanDM to TCGA data reveals 37 pan-cancer DM patterns in the 12 cancer methylomes, including both common and cancer-type-specific patterns. These 37 patterns are in turn used to group cancer types. Functional ontology and biological pathways enriched in the non-common patterns not only underpin the cancer-type-specific etiology and pathogenesis but also unveil the common environmental risk factors shared by multiple cancer types. Moreover, we also identify PanDM-specific DM CpG sites that the common strategy fails to detect.

**Conclusions:**

PanDM is a powerful tool that provides a systematic way to investigate aberrant methylation patterns across multiple cancer types. Results from real data analyses suggest a novel angle for us to understand the common and specific DM patterns in different cancers. Moreover, as PanDM works on the summary statistics for each cancer type, the same framework can in principle be applied to pan-cancer analyses of other functional genomic profiles. We implement PanDM as an R package, which is freely available at http://www.sta.cuhk.edu.hk/YWei/PanDM.html.

## Background

DNA methylation refers to the process of adding methyl groups to DNA segments [1]. As it does not change the nucleic acid of the DNA sequence, it is an epigenetic modification [2]. DNA methylation regulates gene expression [1] and interplays with genetic and environmental alterations [3]. Thus, it has become one of the best characterized epigenetic modifications to date [4, 5]. Aberrant DNA methylation has been confirmed as one of the hallmarks of cancer [6] and has been proposed as a biomarker for cancer prognosis, diagnosis, treatment response, and therapeutic targets [5]. Therefore, to elucidate the cancer mechanism, it is crucial to understand the aberrant DNA methylation patterns across diverse cancer types.

DNA methylation profiles can be measured by both microarray and next-generation sequencing techniques. Microarray platforms such as Illumina Infinium HumanMethylation27 BeadChip and HumanMethylation450 BeadChip measure the methylation level at pre-determined CpG sites [7]. The next-generation sequencing techniques, including whole-genome bisulfite sequencing (WGBS), allow genome-wide profiling of the methylation level at all CpG sites [8]. Nevertheless, due to the cost, the Infinium HumanMethylation450 BeadChip array is still the most commonly adopted for studies with large sample sizes [9].

For microarray and sequencing data, various statistical methods have been proposed to identify CpG sites that show differential methylation (DM) status between case and control samples for a given type of cancer. *IMA* [10], *FastDMA* [11], *Minfi* [12], *MethylMix* [13] can detect DM in array data; for count data, *BSmooth* [14], *MethylKit* [15], *MOABS* [16] and *DSS* [17–19] call DM for sequencing experiments. For cancer studies, another complication is that case samples are often obtained as a mixture of normal cells and cancer cells [20]. Therefore, recently developed DM calling methods also adjust for tumor purity [21, 22]. In this study, we analyze samples assayed by the Infinium HumanMethylation450 BeadChip array, and our model takes tumor-purity-adjusted summary statistics as input data. As our method works on summary statistics, it can also be applied to studies assayed by sequencing technologies as long as the summary statistics that encode DM tendency are provided.

Despite the many single-cancer-based DM calling methods, the common and distinct DM patterns across different cancer types remain elusive. The Cancer Genome Atlas (TCGA) Research Network [23] and the International Cancer Genome Consortium (ICGC) [24] have been collecting multi-omics data for a diverse set of common cancer types over the past several years. The abundant data, particularly the DNA methylation profiles, generated by these large-scale projects offer an unprecedented opportunity to study cancer from a systematic perspective. On one hand, the common DM patterns across cancer types may help to extend the research strategy of studying basic molecular mechanisms and their corresponding effective clinical therapies in well-studied cancer types to other cancer types with similar DM profiles [25]. On the other hand, the DM patterns unique to each cancer type can help to develop novel cancer-type-specific biomarkers. Therefore, pan-cancer DM analysis is crucial for a thorough understanding of cancer etiology.

Recently, several pan-cancer methylation studies have made the first attempts to survey pan-cancer DM patterns. For instance, Kim et al. observed a high level of concordance in the pathways affected by DM genes across different tumor types by investigating 10 distinct cancer methylomes [26]. Gevaert et al. proposed a new method *MethylMix* to identify genes that are DM between normal and disease samples and meanwhile predictive of their own gene expression [27]. The authors applied *MethylMix* to each of 12 cancer methylomes and then studied the DM patterns of “transcriptionally predictive” genes across cancer types [13]. Yang et al. first used limma [28] to identify DM CpG sites for each cancer type individually [29]. Next, they focused on DM CpG sites that are consistently hypermethylated or hypomethylated in at least 8 out of 15 cancer types, as well as those CpG sites that show DM in only a single cancer type [29]. All of these pan-cancer analyses first analyzed each cancer type separately and then directly summarized the findings from separate analyses without a solid statistical model. However, conducting separate analyses in the first stage reduces the statistical power so that weak signals are not detected, which in turn will miss the underlying common and cancer-type-specific DM patterns. Therefore, to fully use the pan-cancer data, joint modeling of DM status across cancer types is urgently needed.

In this article, we propose a novel integrative statistical method named PanDM, which can jointly model DNA methylation data across diverse cancer types by generalizing a meta-analysis method for gene expression data [30]. PanDM assumes that all CpG sites can be divided into several clusters. CpG sites within the same cluster share similar although not identical DM patterns across cancer types. Joint modeling allows DM patterns across cancer types to be learned for each cluster. As a result, the DM status of a given CpG site *g* in a particular cancer type *c* can be determined with reference to its DM status in other cancer types and the DM status of the CpG sites that share the same cluster membership as CpG site *g* in cancer type *c*. Consequently, PanDM offers improved statistical power over the input summary statistics for each separate cancer type. Furthermore, PanDM enables the investigation of biological properties of CpG sites belonging to the same cluster, which are not available with current pan-cancer methylation analyses. We evaluate the performance of PanDM via a simulation study and apply it to the methylomes of 12 cancer types collected from the TCGA project. The results of the enrichment analyses on the clusters learned from the TCGA data suggest that CpG sites with similar pan-cancer patterns indeed share biological implications. In addition, PanDM discovers a set of functional genes missed by the separate analyses.

## Results

### The proposed model

Suppose we want to investigate the differential methylation patterns between normal samples and tumor samples of in total *G* CpG sites across *C* cancer types. Both normal samples and tumor samples are collected for each cancer type. To adjust for the effect of tumor purity, we first call differential methylation for each cancer type separately using InfiniumPurify [22]. InfiniumPurify provides a *p*-value for each CpG site for a given cancer type *c*, *p*_*gc*_, indicating the significance level of DM. As a result, we obtain a matrix ***p***=(*p*_*gc*_)_*G*×*C*_ for all *C* cancer types. Our model aims to learn the pan-cancer-DM patterns from ***p*** and improve DM detection for each cancer type.

**Fig. 1:**
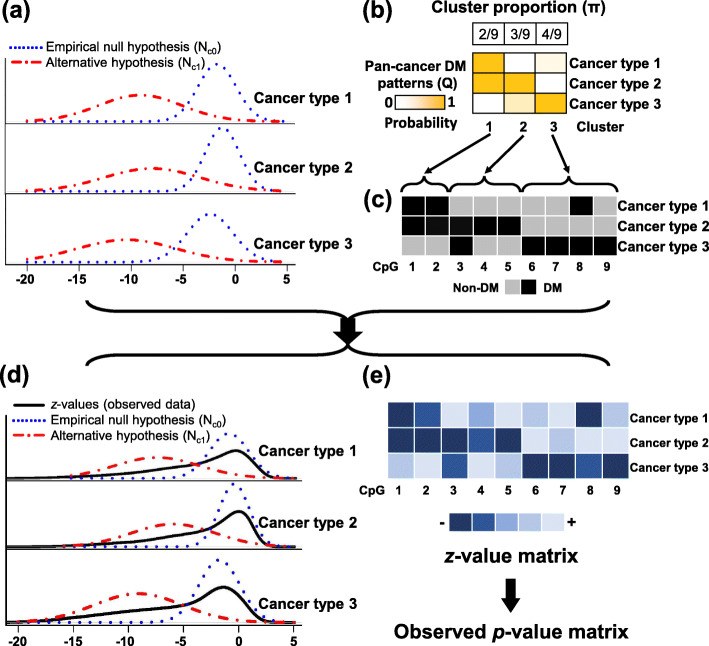
Illustration of the PanDM model. **a** Distributions for the empirical null hypothesis $\left (\mathcal {N}_{c0}\right)$ and the alternative hypothesis $\left (\mathcal {N}_{c1}\right)$ in different cancer types. **b** In this toy example, there are nine CpG sites which can be grouped into three clusters. The cluster proportion corresponds to the ***π*** in our model. The DM tendency in the three cancer types, termed *pan-cancer DM patterns*, are summarized in the probability matrix ***Q*** by PanDM. **c** The DM status matrix ***H*** derived from ***Q***. Each element of ***H*** is a binary variable, indicating whether a specific CpG site is DM or non-DM in every cancer type. **d** Marginal distributions of the observed data. The distribution results from the mixture of $\mathcal {N}_{c0}$ and $\mathcal {N}_{c1}$, where the proportion of each component is given by ***H***. **e** The *z*-values for each CpG site in every cancer type. The *z*-value matrix can be further transformed into the observed *p*-value matrix

We illustrate the PanDM model in Fig. 1. DM detection is a typical large-scale inference problem [31]. For large-scale studies, in contrast to the theoretical null, a more appropriate null can be estimated by leveraging all of the *p*_*gc*_,*g*=1,2,⋯,*G*, for a given cancer type *c*, which is called the “empirical null distribution” [31]. Following the “empirical null approach”, we first transform the *p*-values into *z*-values by *z*_*gc*_=*Φ*^−1^(*p*_*gc*_), where *Φ* is the standard normal cumulative distribution function. Consequently, as shown in Fig. 1a, for each given cancer type, the *Gz*-values *z*_*gc*_,*g*=1,2,⋯,*G*, come from two normal distributions: $\mathcal {N}_{c0}$ for the empirical null hypothesis and $\mathcal {N}_{c1}$ for the alternative hypothesis [31]. For DM detection, the empirical null distribution corresponds to the non-differentially methylated CpG sites, and the alternative represents the DM CpG sites. We denote the underlying true DM status for CpG site *g* in cancer type *c* as *H*_*gc*_, where *H*_*gc*_=1 indicates DM (Fig. 1c). The distribution of *z*_*gc*_ then follows

$$\begin{aligned} \qquad\quad z_{gc}|H_{gc}&=0\sim\mathcal{N}_{c0}\left(x|\mu_{c0},\sigma^{2}_{c0}\right); \\ \qquad\quad z_{gc}|H_{gc}&=1\sim\mathcal{N}_{c1}\left(x|\mu_{c1},\sigma^{2}_{c1}\right). \end{aligned} $$

The parameters of $\mathcal {N}_{c0}$ and $\mathcal {N}_{c1}$ together with *H*_*gc*_ can be learned by fitting two normal mixture distributions to *p*_*gc*_,*g*=1,2,⋯,*G*, for each cancer type individually. Nevertheless, the inference may suffer from low accuracy due to the high level of noise in the methylation data. Therefore, in our proposed model, we attempt to learn the DM patterns across cancer types ***H***_*g*_=(*H*_*g*1_,*H*_*g*2_,…,*H*_*gC*_) together so that the correlations of cancer types help to improve the detection of DM status for each cancer type. This would in turn allow for a better estimation of $\mathcal {N}_{c0}$ and $\mathcal {N}_{c1}$, leading to better DM detection.

Enumerating all combinations of ***H***_*g*_ directly in the model is prohibitive as there are 2^*C*^ possible patterns, which becomes 2^12^=4096 for the 12 cancer types in our analysis. To overcome the exponential growth of the parameter space, we instead assume that all of the CpG sites come from *K* clusters, where *K* is a parsimonious small number compared with 2^*C*^. The CpG sites of the same cluster share similar, although not identical, DM patterns across the *C* cancer types. Specifically, for a CpG site of cluster *k*, denoted as *a*_*g*_=*k*, the probability of DM in cancer type *c* is *q*_*kc*_=Pr(*H*_*gc*_=1|*a*_*g*_=*k*). Consequently, a large *q*_*kc*_ indicates that the CpG sites in cluster *c* are likely to be DM for cancer type *c* (Fig. 1b). Nevertheless, two CpG sites in the same cluster are not required to have exactly the same DM status. In other words, it is not necessary for CpG sites *g* and *g*^′^ within the same cluster to hold $H_{gc}=H_{g^{\prime }c}$ although $\text {Pr}\left (H_{gc}=H_{g^{\prime }c}|a_{g}=a_{g^{\prime }}\right)$ is promoted by our proposed model. Assuming that given the cluster membership *a*_*g*_ the DM status *H*_*gc*_s are independent among different cancer types, then the joint probability of a specific DM configuration ***H***_*g*_ and its corresponding observed ***Z***_*g*_=(*z*_*g*1_,*z*_*g*2_,…,*z*_*gC*_) given that the CpG site belongs to cluster *k* becomes

1 $$ {\begin{aligned} \text{Pr}\left(\boldsymbol{Z}_{g}, \boldsymbol{H}_{g}|a_{g}=k\right)=\prod\limits_{c=1}^{C}\left[q_{kc}\mathcal{N}_{c1}\left(z_{gc}\right)\right]^{H_{gc}}\left[\left(1-q_{kc}\right)\mathcal{N}_{c0}\left(z_{gc}\right)\right]^{\bar{H}_{gc}}, \end{aligned}}  $$

where $\bar {H}_{gc}=1-H_{gc}$.

Denote the prevalence of cluster *k* among all of the CpG sites as *π*_*k*_ and collect ***Z***=(*z*_*gc*_)_*G*×*C*_, ***A***=(*a*_1_,…,*a*_*G*_), ***π***=(*π*_1_,*π*_2_,…,*π*_*K*_), ***Q***=(*q*_*kc*_)_*K*×*C*_, ***μ***=(*μ*_10_,…,*μ*_*C*0_,*μ*_11_,…,*μ*_*C*1_) and ***Σ***=(*σ*_10_,…,*σ*_*C*0_,*σ*_11_,…,*σ*_*C*1_). The joint distribution can be written as:

2 $$ {\begin{aligned}\text{Pr}\left(\boldsymbol{Z},\boldsymbol{H},\boldsymbol{A}|\boldsymbol{\pi},\boldsymbol{Q},\boldsymbol{\mu},\boldsymbol{\Sigma}\right)=\prod\limits_{g=1}^{G}\prod\limits_{k=1}^{K}\left\{\pi_{k}\text{Pr}\left(\boldsymbol{Z}_{g}, \boldsymbol{H}_{g}|a_{g}=k\right)\right\}^{I\left(a_{g}=k\right)}, \end{aligned}}  $$

where *I*(·) is the indicator function with *I*(*S*)=1 if *S* is true and *I*(*S*)=0 otherwise.

We collect the model parameters into ***Θ***={***π***,***Q***,***μ***,***Σ***}. Note that in the above joint distribution, only ***Z*** are observed data. Both ***H*** and ***A*** are latent variables. Thus, PanDM adopts the expectation-maximization (EM) algorithm [32] to estimate ***Θ***. The optimal number of clusters is determined by the Bayesian information criterion (BIC) [33]. The derivation of the parameter inferences, pattern number selection, and DM status identification are detailed in the “Methods” section. PanDM is implemented as an R package and is available at http://www.sta.cuhk.edu.hk/YWei/PanDM.html.

### Simulation

**Fig. 2:**
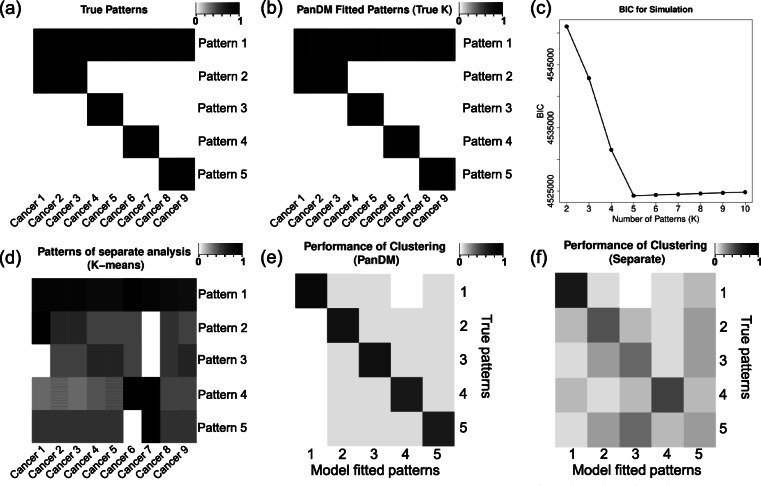
Simulation results. **a** The assumed underlying true DM patterns *Q*. Each row indicates one of the five patterns; each column corresponds to one cancer type. A darker color suggests a higher probability of being DM. **b** Detailed DM patterns of the five clusters $\hat {Q}$ learned by PanDM, which almost exactly match the true patterns. **c** The BIC plot for PanDM from *K*=2 to *K*=20. PanDM achieves the minimal BIC at the assumed true *K*=5. **d** The five DM patterns learned by separate analysis from the simulation data, which are prone to noise and deviate from the underlying true patterns. **e** Pattern matching matrix of PanDM and the assumed true cluster labels. The color in cell (*k*,*l*) corresponds to the number of CpG sites identified as model-fitted pattern *l* by PanDM while actually belonging to pattern *k* normalized by the total number of true cluster *k* CpG sites. Thus, the diagonal suggests the proportion of matched cases. From the heatmap we can see that PanDM correctly groups most of the CpG sites. **f** Pattern matching matrix from the separate analysis and the assumed true cluster labels. The vague diagonal indicates the high misclassification rate of the separate analysis

We evaluate the performance of PanDM via a simulation study. The synthetic data are generated as follows. We assume that in total *G*=100,000 CpG sites are measured for *C*=9 cancer types. There are *K*=5 distinct DM patterns among all of the *G* CpG sites. We randomly choose a *π*_*k*_ proportion of CpG sites to belong to pattern *k*, where 0<*π*_*k*_<1, and ${\sum \nolimits }_{k=1}^{5}\pi _{k}=1$. *a*_*g*_=*k* indicates that CpG site *g* belongs to pattern *k*. For DM pattern *k*, the probability of DM in cancer type *c* is equal to *q*_*kc*_. The matrix ***Q***=(*q*_*kc*_)_*K*×*C*_, shown in Fig. 2a, summarizes the DM patterns across all cancer types (see Additional file 1, Table S1 for numerals). The DM status *H*_*gc*_ for a given CpG site *g* in cancer type *c* is sampled as a Bernoulli random variable *Ber*(*q*_*kc*_). If *H*_*gc*_=1, which means that CpG site *g* is DM in cancer type *c*, then its *z*-value *Z*_*gc*_ is generated from $\mathcal {N}\left (\mu _{c1},\sigma ^{2}_{c1}\right)$; if CpG site *g* is not DM in cancer type *c*, i.e. *H*_*gc*_=0, then *Z*_*gc*_ follows $\mathcal {N}\left (\mu _{c0},\sigma ^{2}_{c0}\right)$. The specific settings for $\mu _{c1},\mu _{c2},\sigma ^{2}_{c1},\sigma ^{2}_{c2}$ are listed in Additional file 1, Table S2. Consequently, we obtain the *z*-value matrix **Z**.

We apply PanDM to **Z**. We set the tolerance bound *ε* for ∥***Θ***^(*n*+1)^−***Θ***^(*n*)^∥ to 1e-4 and let *K* vary from 2 to 10. According to the BIC plot in Fig. 2c, the lowest BIC value is reached at *K*=5. Therefore, PanDM recovers the true number of assumed DM patterns. Moreover, Fig. 2b shows that the estimated $\boldsymbol {\hat {Q}}=\left (\hat {q}_{kc}\right)_{K\times C}$ matches exactly with the underlying true DM patterns ***Q*** shown in Fig. 2a.

**Fig. 3:**
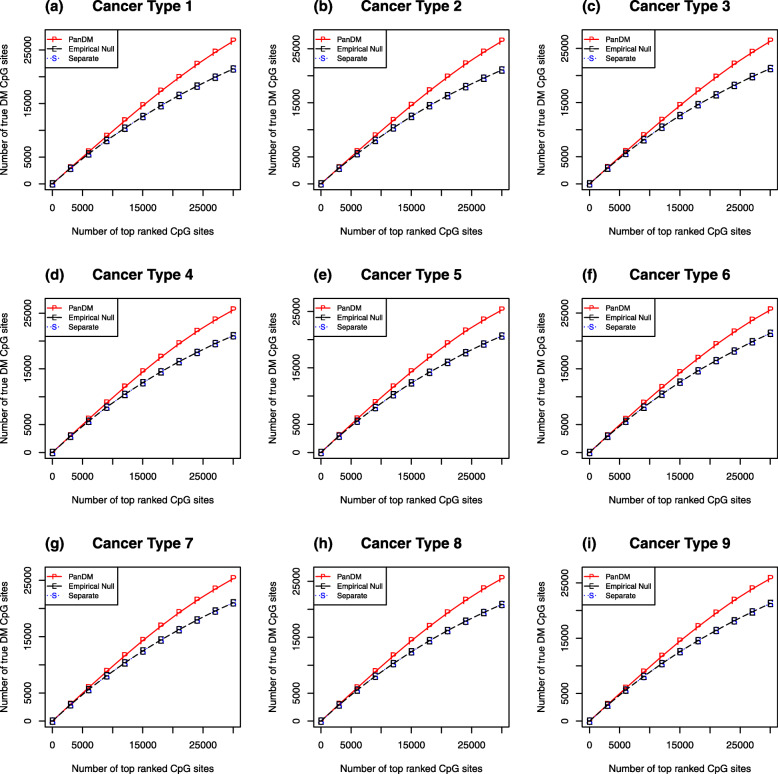
Comparison of model performance. (a-i) The number of true positives among the top-ranked CpG sites by each of the three DM calling methods. “P” refers to PanDM; “E” refers to “Empirical Null”, corresponding to the strategy of fitting two-normal mixtures to the single-study-based *p*-values of each cancer type individually; “S” indicates our separate analyses based on the rank of the *p*-values. The two separate analyses produce almost the same results when detecting top-ranked CpG sites. PanDM identifies more true positive DM CpG sites

We compare the DM calling performance of PanDM with that of two types of separate analyses. For Type I separate analyses, we simply rank the CpG sites for each cancer type separately according to their *p*-values transformed from the corresponding *z*-values. This type of analysis corresponds to the widely adopted practice in EWAS studies. For Type II separate analyses, we fit the “empirical null” to the *z*-values [31]. Specifically, we fit a mixture model with two normal components to the data for each cancer type separately using the EM algorithm. Then, we rank the CpG sites according to their probabilities of belonging to the non-null component. This allows us to investigate where the power of PanDM lies. For each of the three methods, we count the number of true positives among the top-ranked CpG sites. From Fig. 3a-i, we can see that the performance of the Type II separate analyses is about the same as that of the Type I separate analyses. PanDM, however, beats both types of separate analyses. Therefore, the improvement in PanDM’s power to detect DM mainly arises from joint modeling across different cancer types rather than from the “empirical null” approach.

Next we compare the performace of PanDM and separate analyses on the clustering of DM patterns. We focus on Type I separate analyses because this type of strategy is the common practice in current studies. We control the global false discovery rate (FDR) of the simulated *p*-values at 0.01 and assign a binary state, denoted as *ρ*_*gc*_, to each CpG site to indicate its DM status in cancer type *c*. We regard the indicator vector ***ρ***_***g***_=(*ρ*_*g*1_,*ρ*_*g*2_,⋯,*ρ*_*g*9_) as the pan-cancer DM pattern for CpG site *g* resulting from separate analyses. We then apply K-means clustering [34] to the DM patterns of all 100,000 CpG sites and group them into 5 clusters. For each cluster, we calculate the group mean $\boldsymbol {\hat {\rho }_{k}}=mean_{g\in group_{k}}\left (\boldsymbol {\rho _{g}}\right)$. Figure 2d shows $\boldsymbol {\hat {\rho }_{k}}, k=1,2,\cdots, 5$. Compared with Fig. 2a and b, the separate analysis fails to identify the true underlying DM patterns across the cancer types and is prone to noise. Moreover, from PanDM, we can determine which DM pattern each CpG site belongs to according to $\text {Pr}\left (a_{g}=k|\boldsymbol {Z},\hat {\boldsymbol {\Theta }}\right)$. Figure 2e presents the pattern matching matrix, which demonstrates the accuracy of PanDM’s DM pattern classification. In contrast, Fig. 2f is the corresponding pattern matching matrix for the Type II separate analyses, which has a much higher misclassification error rate.

We further investigate the scenario where PanDM is applied to the dataset with a pre-specified cluster number $\hat {K}$ that differs from the true underlying *K*. Suppose that $\hat {K}$ is set to 4, which is smaller than the true cluster number *K*=5. Then, the true underlying patterns 4 and 5 are learned as a single merged pattern 4 for $\hat {K}=4$, as shown in Additional file 1, Fig. S1. Nevertheless, patterns 1-3 are learned the same under both scenarios. In contrast, when $\hat {K}>K$, the original pattern 2 is split into two separate new patterns, while the other DM patterns stay the same (see Additional file 1, Fig. S2). Therefore, the DM pattern matrix $\hat {Q}$ can reflect the underlying DM pattern even when the number of clusters $\hat {K}$ is mis-specified. Furthermore, we evaluate the capability of PanDM to identify the true positives when $\hat {K}$ deviates from the underlying *K*. Figs. S3 and S4 in Additional file 1 demonstrate that PanDM still outperforms both types of separate analyses. Therefore, even when *K* is not searched exactly, PanDM still provides a legitimate estimation of the DM patterns and improves the detection power.

In summary, the simulation study illustrates that PanDM can accurately estimate the model parameters, evaluate the global FDR, determine the DM status, identify the DM patterns across cancer types and cluster CpG sites according to their DM patterns.

### Application to TCGA data

**Model-fitting results** We downloaded the methylomes of 12 cancer types from the TCGA project [23]. We first call DM for each cancer type by InfiniumPurify [22] adjusting for the effects of tumor purity. We then transform the obtained *p*-values into corresponding *z*-values and apply PanDM to the *z*-value matrix. The chosen number of candidate patterns K ranges from 5 to 50. According to the BIC plot shown in Additional file 1, Fig. S5, the optimal number of pan-cancer DM patterns is *K*=37. Given $\hat {K}=37$, we first evaluate how well PanDM fits the real data. Specifically, for each cancer type, we generate random samples from the mixture distributions with the parameters $\hat {\pi }_{k},\hat {q}_{kc},\hat {\mu }_{jc},\hat {\sigma }_{jc}$, and then produce the quantile-quantile (Q-Q) plots for the samples against the real observed data (see Additional file 1, Fig. S6). These Q-Q plots suggest that our estimated mixture distributions closely match the marginal distributions of the real data. Therefore, PanDM fits the real data well. We provide the PanDM cluster membership for each CpG site in Additional file 2, Table S4.

**Fig. 4:**
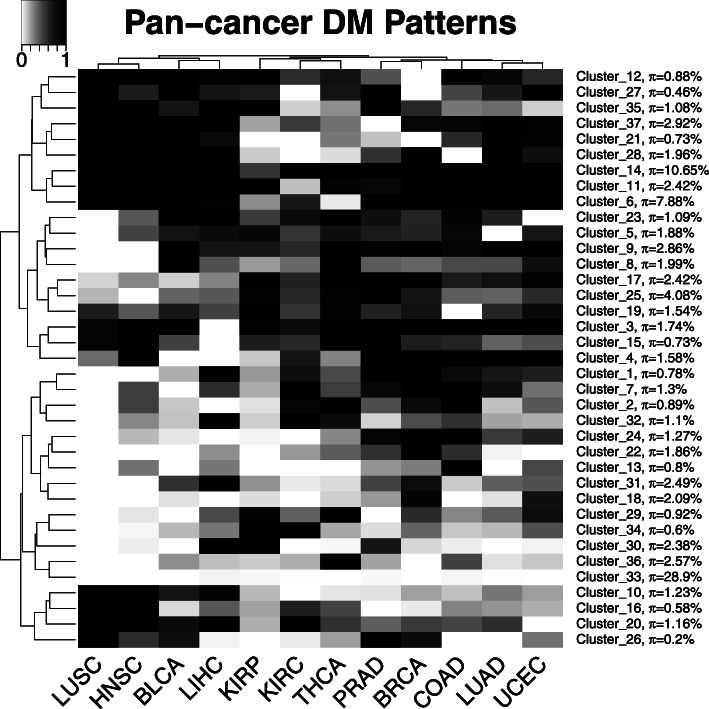
The detailed DM patterns across the 12 cancer types learned by PanDM. The proportion of each pattern, *π*_*k*_, is also given next to the cluster label. The darker the color in the cells of the heatmap, the higher the probability of DM

Figure 4 shows the detailed 37 DM patterns and their proportions. The largest among all of the learned clusters is cluster 33, which represents the non-DM pattern in the 12 investigated cancer types. Therefore, a large proportion of CpG sites are not affected by cancer. The second largest cluster, cluster 14, characterizes the DM pattern in all the 12 investigated cancer types. Previous studies have mainly focused on this type of consistent DM pattern [23, 26, 29]. Nevertheless, PanDM reveals that many DM patterns are cancer-type dependent.

The pan-cancer DM patterns are also helpful for grouping cancer types. We apply hierarchical clustering with the complete linkage method and Euclidean distance to both DM patterns and cancer types. In Fig. 4, the 12 cancer types are classified into 5 subgroups according to the hierarchical clustering tree. The patterns in the LUSC-HNSC group show high concordance, suggesting the epigenetic commonality of squamous cell carcinoma. Tracing back to the root node of the clustering tree, LUSC, HNSC, BLCA, and LIHC can be further grouped together, which is consistent with the previous finding that LUSC, HNSC and BLCA are squamous-like subtypes [35]. LUSC and LUAD, despite being the two main subtypes of non-small-cell lung carcinoma [36], have distinct disease methylomes according to our PanDM results. Thus, these two cancer types are distant from each other in the clustering tree in Fig. 4. The grouping of cancer types by pan-cancer DM patterns provides a novel perspective from which to study the epigenetic similarities between different tumor types.

**Biological interpretation** To further understand the biological implications of the pan-cancer DM patterns, we conduct enrichment analyses for all of the CpG sites in each of the 37 clusters using the GREAT tool [37] with the default parameters. We examine the enrichments of “Gene Ontology (GO)”, “Disease Ontology”, “MSigDB/ PANTHER/ BioCyc Pathway” and “MSigDB Cancer Neighborhood” (see Additional file 3, Table S5A and Additional file 4, Table S5B).

We first investigate Cluster 33, the all-non-DM pattern. It is enriched with several essential biological processes and pathways, including “nuclear-transcribed mRNA catabolic process”, “translational initiation”, “translational elongation”, “genes involved in metabolism of RNA”, “genes involved in transcription” and “genes involved in mRNA splicing” (see Additional file 3, Table S5A). All of these processes and pathways are responsible for maintaining basic biological functions in the human body. We expect the majority of genes involved in these processes and pathways to function properly despite the occurrence of cancers, which is consistent with our discovered all-non-DM pattern.

To consider the enriched ontology terms with strong signals, for the remaining 36 clusters, in addition to the 0.05 FDR threshold, we add another filtering criterion requiring fold enrichment to be larger than two. Only those ontology terms that pass both criteria are recorded (see Additional file 4, Table S5B) and discussed in the following.

Under the more stringent criterion, functional significance is still found for several clusters. Cluster 13 is mainly composed of CpG sites that are only DM in COAD. One of the enriched ontology terms for this cluster is the biological process “intestinal epithelial cell differentiation”. It has been reported that CDX-2, a transcription factor involved in the proliferation and differentiation of intestinal epithelial cells, is an important biomarker for colon adenocarcinoma [38]. This suggests that the enriched biological process is indeed closely related to colon cancer. Thus, cluster 13 can help us to identify more COAD-specific DM genes that contribute to the carcinogenesis of colorectal cancer.

Cluster 16 presents a pattern of strong DM tendency in LUSC and HNSC according to our PanDM analyses (Fig. 4). One of its enriched “GO Biological Process” terms is “response to UV-B”. Ultraviolet B (UVB) is one of the major carcinogens involved in squamous cell skin cancers [39]. The enrichment of UVB-response-related biological processes suggests that this cluster contains genes that are commonly affected in squamous cell carcinoma. We expect that cluster 16 can be used to discover potential squamous cell carcinoma-specific biomarkers or therapeutic targets.

Cluster 18 captures the pattern of DM in two gender-specific cancer types: BRCA and UCEC. A closer inspection reveals that this cluster includes more X-chromosome-located CpG sites than any other cluster except cluster 33 (see Additional file 5, Table S6). It has been observed that uterine serous carcinomas and basal-like breast carcinomas share many molecular features, including similar DNA methylation alterations [40]. Therefore, cluster 18 supports previous findings and can serve as a useful resource for further exploration of the underlying relationship between breast and endometrial cancers. This cluster is also enriched with the biological process “ubiquitin-dependent SMAD protein catabolic process”. It has been reported that hyperactivity of the SMAD signaling pathway is required to maintain the epigenetic silencing of epithelial-mesenchymal transition genes during breast cancer progression [41]; therefore, we expect that cluster 18 collects genes that contribute to the activation of the SMAD signaling pathway.

Cluster 22 suggests another BRCA-specific DM pattern. However, its CpG sites tend to be non-DM in UCEC. Among the significantly enriched ontology terms for this cluster, two MSigDB pathways are notable: “Genes involved in Class B/2 (secretin family receptors)” and the “Hedgehog signaling pathway”. Dysregulated secretin receptors have been linked to aberrant methylation in breast cancer tissues [42]. Meanwhile, the Hedgehog signaling pathway also plays an essential role in the development of breast cancer [43] and is now considered as a potential anticancer target [44]. These facts confirm the BRCA-specific DM pattern of cluster 22.

In addition to the patterns with DM specificity in only one or two cancer types, PanDM detected DM in a large number of cancer types. Cluster 28 encompasses CpG sites with a strong DM tendency in 6 out of the 12 investigated cancer types: LUSC, HNSC, BLCA, LIHC, PRAD and LUAD. This cluster is enriched with three MSigDB pathways: “genes involved in presynaptic nicotinic acetylcholine receptors”, “genes involved in acetylcholine binding and downstream events”, and “genes involved in highly calcium-permeable postsynaptic nicotinic acetylcholine receptors”. These pathways are involved in tobacco-induced carcinogenesis because nicotine, the principle component of cigarette, can stimulate cell proliferation as well as facilitate tumor growth and survival by binding to nicotinic acetylcholine receptors (nAChRs) [45]. Hence, the enrichment of nAChR-related pathways suggests that cluster 28 contains CpG sites whose methylation status is commonly altered in cancers induced by tobacco carcinogens. Moreover, the six cancer types with a strong DM tendency in this pattern are more likely to be associated with cigarette smoking than the remaining six. In fact, smoking increases the risk of lung cancers (LUAD, LUSC) [46], liver cancer (LIHC) [47], cancer of the oral cavity (HNSC) [48] and bladder cancer (BLCA) [49].

Apart from the biological interpretation of the pan-cancer DM patterns, we investigate whether PanDM performs better than traditional separate analyses on the real data. We again adopt Type I separate analyses for benchmarking. Controlling global FDRs at 0.01, we obtain two sets of dichotomous classification (DM/non-DM) for all of the CpG sites in each cancer type. CpG sites that are identified as non-DM by separate analyses but as DM by PanDM are defined as *PanDM-specific DM CpG sites (PanDM-specific DMC)*. The numbers of *PanDM-specific DMC* vary across the 12 different cancer types (see Additional file 1, Fig. S7). We focus on the results from UCEC, as it has the largest number of *PanDM-specific DMC*. Most of the 3,094 UCEC *PanDM-specific DMC* come from pan-cancer DM patterns 6 and 14. These CpG sites can be mapped to 1,285 unique genes. As multiple CpG sites can correspond to one single gene on the Infinium HumanMethylation450 BeadChip array, we remove the genes that match at least one DM CpG site identified by separate analyses. We find three UCEC *PanDM-specific DMC* genes that are directly associated with cancer according to the KEGG pathway annotation by DAVID [50, 51]: *EI24*, *GNGT2*, and *MIR21*. *EI24* encodes an autophagy-associated transmembrane protein, which is a putative tumor suppressor due to its role as a downstream induction target of p53-dependent apoptosis [52]. Its genomic location, chromosome 11q24, is also a region with frequent mutation in cancer cases [53, 54]. *GNGT2* encodes a transducin that may be involved in many cancer-related pathways such as the “chemokine signaling pathway” and “PI3K-Akt signaling pathway” [55]. *MIR21* encodes an important microRNA, miR-21, in mammal. It is one of the frequently dysregulated microRNAs in cancer and most of its targets are tumor suppressors [56]. According to these well-established functions of *EI24*, *GNGT2* and *MIR21*, PanDM’s identification of their DM status in cancers is highly likely to reflect a biological reality. All of these results demonstrate that PanDM can help to retrieve DM signals missed by separate analyses.

## Discussion

In this paper, we propose PanDM, an integrative statistical model that can learn DM patterns across diverse cancer types and thereby improve DM detection for each cancer type. Previous methods call DM separately for each cancer type and then focus on the identified DM CpG sites with strong signals from each cancer type. However, the first stage of individual screening for DM CpG sites not only is likely to miss those weak signals, but also fails to fully use the information from those non-DM CpG sites that may be helpful for DM detection in other cancer types. For instance, the pan-cancer DM pattern 26 learned from the TCGA dataset tends to be totally non-DM in KIRP, COAD and LUAD but has a high DM preference in LUSC and PRAD. Therefore, for a CpG site that belongs to this pattern, if we are uncertain about its DM status in PRAD but are sure that it is non-DM in KIRP, COAD and LUAD as well as DM in LUSC, then we can be more confident in claiming DM for it in PRAD. Hence, PanDM fully uses the information across cancer types to improve signal detection. Consequently, PanDM offers a more accurate and comprehensive picture of DM status for all the measured CpG sites across all investigated cancer types simultaneously.

Currently, PanDM works on summary statistics from each cancer type following the “empirical null” approach [31]. As a result, PanDM accepts the output of any single-cancer-type-based DM calling method as long as it provides a list of *p*-values. Therefore, any advance in single-cancer-based DM method can be conveniently incorporated right away. For instance, in this paper, we adopt the tumor-purity-adjusted DM calling method. Meanwhile, now we follow the tradition of the “empirical null” to fit a two Gaussian mixture to the summary statistics, one for the null and the other for the alternative, which has been shown to be very effective for most high-dimensional genomic datasets [57]. In principle, we can also fit a three-component Gaussian mixture to further discriminate between hypo-methylation and hyper-methylation. PanDM can easily handle such generalization straightforward. Nevertheless, to allow the flexibility to work with any DM calling method where usually only *p*-values are provided, at present we focus on the classic “empirical null” approach to distinguish between DM and non-DM only. Users can further plot heatmaps for each DM pattern to explore the direction of aberrant DNA methylation. Moreover, the current approach enables PanDM to be applied to pan-cancer analyses of other types of functional genomic assays such as gene expression, SNP data, and copy number variation detection as long as *p*-values for each individual cancer type are provided. We foresee that such flexibility will greatly advance pan-cancer analyses.

PanDM clusters CpG sites according to their DM patterns across cancer types. CpG sites assigned the same cluster membership share similar DM patterns. Therefore, they are likely to be driven by the same underlying biological mechanism. Our pathway and ontology enrichment results for the 37 clusters learned from the TCGA data suggest that these pan-cancer DM patterns indeed have distinct biological implications. PanDM provides not only a more accurate way to identify DM CpG sites but also a novel clustering strategy for pan-cancer DM analysis. Different DM patterns will be helpful for oncologists to obtain a comprehensive picture of the mechanisms and etiologies of cancers. Moreover, the clustering analysis suggests that it would be better to select CpG sites from different clusters rather than the same cluster for future biomarker discovery, as CpG sites from diverse clusters provide richer non-redundant information in describing the DM patterns.

We believe that PanDM will greatly advance our understanding of the shared molecular mechanisms in distinct cancer types, help us to identify the unique features of each cancer type, and help us to discover new cancer-type-specific biomarkers. We hope PanDM will become an indispensable tool for pan-cancer analyses.

## Conclusion

Pan-cancer analyses provide an efficient means of learning the common and varied characteristics shared by distinct tumor types. Both similarities and differences between cancer types can guide us to find better clinical therapies. Despite the rapid accumulation of cancer genomic profiles in the public data repositories, comprehensive and systematic pan-cancer analyses are still limited due to the lack of rigorous statistical methods. In this work, we develop a novel model, PanDM, for pan-cancer methylome analysis. PanDM facilitates the joint analysis of multiple distinct cancer methylation profiles and enhances DM signal detection. In both the simulation study and real data analysis, PanDM outperforms the traditional method and offers a new perspective for pan-cancer DM patterns with novel biological insights.

## Methods

**Parameter estimation by PanDM** According to the joint distribution in (2), the complete log-likelihood function is

3 $$ {}\begin{aligned} &\text{ln}L_{comp}(\boldsymbol{\Theta}|\boldsymbol{Z},\boldsymbol{H},\boldsymbol{A})=\sum\limits_{g=1}^{G}\sum\limits_{k=1}^{K}I\left(a_{g}=k\right)\text{ln}\pi_{k} \\ &+\sum\limits_{g=1}^{G}\sum\limits_{k=1}^{K}I\left(a_{g}=k\right)\sum\limits_{c=1}^{C}H_{gc}\left[\text{ln}q_{kc}+\text{ln}\mathcal{N}_{c1}\left(z_{gc}\right)\right] \\ &+\sum\limits_{g=1}^{G}\sum\limits_{k=1}^{K}I\left(a_{g}=k\right)\sum\limits_{c=1}^{C}\bar{H}_{gc}\left[\text{ln}(1-q_{kc})+\text{ln}\mathcal{N}_{c0}(z_{gc})\right]. \end{aligned}  $$

In the *n*-th iteration of the EM algorithm, we denote the current parameter estimates as ***Θ***^(*n*)^ and derive the following E-step and M-step.

In the E-step, we calculate the *Q*-function, the conditional expectation of the log-likelihood, as follows:

4 $$ {\begin{aligned} &Q\left(\boldsymbol{\Theta}|\boldsymbol{\Theta}^{(n)}\right)=\mathrm{E}\left[\text{ln}L_{comp}|\boldsymbol{Z},\boldsymbol{\Theta}^{(n)}\right] \\ =&\sum\limits_{g=1}^{G}\sum\limits_{k=1}^{K}\left(\text{ln}\pi_{k}\right)\mathrm{E}\left[I\left(a_{g}=k\right)|\boldsymbol{Z},\boldsymbol{\Theta}^{(n)}\right] \\ +&\sum\limits_{g=1}^{G}\sum\limits_{k=1}^{K}\sum\limits_{c=1}^{C}\left\{\left[\text{ln}q_{kc}+\text{ln}\mathcal{N}_{c1}\left(z_{gc}\right)\right]\mathrm{E}\left[I\left(a_{g}=k\right)H_{gc}|\boldsymbol{Z},\boldsymbol{\Theta}^{(n)}\right]\right. \\ &\left.+\left[\text{ln}\left(1-q_{kc}\right)+\text{ln}\mathcal{N}_{c0}\left(z_{gc}\right)\right]\mathrm{E}\left[I\left(a_{g}=k\right)\bar{H}_{gc}|\boldsymbol{Z},\boldsymbol{\Theta}^{(n)}\right]\right\}. \end{aligned}}  $$

Here, the conditional expectation for the cluster membership of a CpG site *g* is calculated as

5 $$ {\begin{aligned} &\mathrm{E}\left[I\left(a_{g}=k\right)|\boldsymbol{Z},\boldsymbol{\Theta}^{(n)}\right]=\text{Pr}\left(a_{g}=k|\boldsymbol{Z},\boldsymbol{\Theta}^{(n)}\right) \\ &=\frac{\text{Pr}\left(\boldsymbol{Z}|a_{g}=k,\boldsymbol{\Theta}^{(n)}\right)\text{Pr}\left(a_{g}=k,\boldsymbol{\Theta}^{(n)}\right)}{{\sum\nolimits}_{j=1}^{K}\text{Pr}\left(\boldsymbol{Z}|a_{g}=j,\boldsymbol{\Theta}^{(n)}\right)\text{Pr}\left(a_{g}=j,\boldsymbol{\Theta}^{(n)}\right)} \\ &=\frac{\pi_{k}^{(n)}\prod\nolimits_{c=1}^{C}\left[q_{kc}^{(n)}\mathcal{N}_{c1}^{(n)}\left(z_{gc}\right)+\left(1-q_{kc}^{(n)}\right)\mathcal{N}_{c0}^{(n)}\left(z_{gc}\right)\right]}{{\sum\nolimits}_{j=1}^{K}\pi_{j}^{(n)}\prod\nolimits_{c=1}^{C}\left[q_{jc}^{(n)}\mathcal{N}_{c1}^{(n)}\left(z_{gc}\right)+\left(1-q_{jc}^{(n)}\right)\mathcal{N}_{c0}^{(n)}\left(z_{gc}\right)\right]}, \end{aligned}}  $$

where $\mathcal {N}_{c1}^{(n)}\left (z_{gc}\right)=\mathcal {N}_{c1}\left (z_{gc}|\mu _{c1}^{(n)},\sigma _{c1}^{(n)}\right)$ and the same abbreviation applies to $\mathcal {N}_{c0}^{(n)}\left (z_{gc}\right)$.

As Eq. (5) shows, the cluster membership for CpG site *g* is determined by comparing the likelihood of its observed *p*-values across all cancer types under the DM patterns of each cluster. Subsequently, information is pooled over cancer types.

Meanwhile, the conditional probability of DM for CpG site *g* given that it belongs to *k* becomes

6 $$ {\begin{aligned} &\mathrm{E}\left[I\left(a_{g}=k\right)H_{gc}|\boldsymbol{Z},\boldsymbol{\Theta}^{(n)}\right]=\text{Pr}\left(a_{g}=k,H_{gc}=1|\boldsymbol{Z},\boldsymbol{\Theta}^{(n)}\right) \\ &=\text{Pr}\left(H_{gc}=1|a_{g}=k,\boldsymbol{Z},\boldsymbol{\Theta}^{(n)}\right)\text{Pr}\left(a_{g}=k|\boldsymbol{Z},\boldsymbol{\Theta}^{(n)}\right) \\ &=\frac{q_{kc}^{(n)}\mathcal{N}_{c1}^{(n)}\left(z_{gc}\right)\text{Pr}\left(a_{g}=k|\boldsymbol{Z},\boldsymbol{\Theta}^{(n)}\right)}{q_{kc}^{(n)}\mathcal{N}_{c1}^{(n)}\left(z_{gc}\right)+\left(1-q_{kc}^{(n)}\right)\mathcal{N}_{c0}^{(n)}\left(z_{gc}\right)}. \end{aligned}}  $$

As Eq. (6) involves $q_{kc}^{(n)}$, the DM status of CpG site *g* in cancer type *c* borrows strengths from the DM status of other CpG sites in cluster *k*, and thus is more robust to noise.

In the M-step, we maximize the *Q*-function with respect to ***Θ*** and obtain new parameter estimates:

7) (8) (9 $$\begin{array}{*{20}l} {}\pi_{k}^{(n+1)}&=\frac{{\sum\nolimits}_{g=1}^{G}\mathrm{E}\left[I\left(a_{g}=k\right)|\boldsymbol{Z},\boldsymbol{\Theta}^{(n)}\right]}{G}, \\ {}q_{kc}^{(n+1)}&=\frac{{\sum\nolimits}_{g=1}^{G}\mathrm{E}\left[I\left(a_{g}=k\right)H_{gc}|\boldsymbol{Z},\boldsymbol{\Theta}^{(n)}\right]}{{\sum\nolimits}_{g=1}^{G}\mathrm{E}\left[I\left(a_{g}=k\right)|\boldsymbol{Z},\boldsymbol{\Theta}^{(n)}\right]}, \\ {}\mu_{c1}^{(n+1)}&=\frac{{\sum\nolimits}_{g=1}^{G}z_{gc}\mathrm{E}\left[I\left(a_{g}=k\right)H_{gc}|\boldsymbol{Z},\boldsymbol{\Theta}^{(n)}\right]}{{\sum\nolimits}_{g=1}^{G}\mathrm{E}\left[I\left(a_{g}=k\right)H_{gc}|\boldsymbol{Z},\boldsymbol{\Theta}^{(n)}\right]}, \end{array} $$

10 $$ {\begin{aligned} \left(\sigma_{c1}^{(n+1)}\right)^{2}&=\frac{{\sum\nolimits}_{g=1}^{G}\left(z_{gc}-\mu_{c1}^{(n+1)}\right)^{2}\mathrm{E}\left[I\left(a_{g}=k\right)H_{gc}|\boldsymbol{Z},\boldsymbol{\Theta}^{(n)}\right]}{{\sum\nolimits}_{g=1}^{G}\mathrm{E}\left[I\left(a_{g}=k\right)H_{gc}|\boldsymbol{Z},\boldsymbol{\Theta}^{(n)}\right]}, \end{aligned}}  $$

11 $$\begin{array}{*{20}l} {}\mu_{c0}^{(n+1)}&=\frac{{\sum\nolimits}_{g=1}^{G}z_{gc}\mathrm{E}\left[I\left(a_{g}=k\right)\bar{H}_{gc}|\boldsymbol{Z},\boldsymbol{\Theta}^{(n)}\right]}{{\sum\nolimits}_{g=1}^{G}\mathrm{E}\left[I\left(a_{g}=k\right)\bar{H}_{gc}|\boldsymbol{Z},\boldsymbol{\Theta}^{(n)}\right]}, \end{array} $$

12 $$ {\begin{aligned} \left(\sigma_{c0}^{(n+1)}\right)^{2}&=\frac{{\sum\nolimits}_{g=1}^{G}\left(z_{gc}-\mu_{c0}^{(n+1)}\right)^{2}\mathrm{E}\left[I\left(a_{g}=k\right)\bar{H}_{gc}|\boldsymbol{Z},\boldsymbol{\Theta}^{(n)}\right]}{{\sum\nolimits}_{g=1}^{G}\mathrm{E}\left[I\left(a_{g}=k\right)\bar{H}_{gc}|\boldsymbol{Z},\boldsymbol{\Theta}^{(n)}\right]}. \end{aligned}}  $$

The two steps are iterated until ∥***Θ***^(*n*+1)^−***Θ***^(*n*)^∥ is smaller than a pre-specified error tolerance bound *ε*.

We denote the estimates obtained from the EM algorithm as $\hat {\boldsymbol {\Theta }}\,=\,\left \{\hat {\pi }_{k},\hat {q}_{kc},\hat {\mu }_{jc},\hat {\sigma }_{jc}:k\,=\,1,\cdots,K;c=1,\cdots,C;j=0,1\right \}$.

**Pattern number selection** PanDM adopts the BIC to determine the number of DM patterns *K*. Specifically, for a given *K*, we calculate the BIC as

13 $$ \begin{aligned} \text{BIC}(K)=&-2\text{ln}\hat{L}_{obs}+(K-1+KC+4C)\text{ln}G \\ =&-2\sum\limits_{g=1}^{G}\text{ln}\sum\limits_{k=1}^{K}\left\{\hat{\pi}_{k}\prod\limits_{c=1}^{C}\left[\hat{q}_{kc}\mathcal{N}_{c1}\left(z_{gc}|\hat{\mu}_{c1},\hat{\sigma}^{2}_{c1}\right)\right.\right. \\ &\left.\left.+\left(1-\hat{q}_{kc}\right)\mathcal{N}_{c0}\left(z_{gc}|\hat{\mu}_{c0},\hat{\sigma}^{2}_{c0}\right)\right]{\vphantom{\frac{1}{\frac{1}{2}}}}\right\} \\ &+(K-1+KC+4C)\text{ln}G. \end{aligned}  $$

The BIC values for different *K*s are evaluated, and the one with the smallest BIC is chosen as the optimal *K*, denoted as $\hat {K}$.

**DM pattern classification** Once the number of DM patterns *K* is determined, the DM pattern for the *k*^*th*^ group is estimated as $\hat {Q}_{k}=\left (\hat {q}_{k1},\hat {q}_{k2},\ldots,\hat {q}_{kC}\right)$. $\hat {q}_{kc}$ represents the probability that a CpG site belongs to group *k* and is DM in cancer type *c*. For a given CpG site *g*, it is classified as belonging to group $k_{g}=max_{k}\left \{\text {Pr}\left (a_{g}=k|\boldsymbol {Z},\hat {\boldsymbol {\theta }}\right)\right \}$, and its DM pattern is classified as that of group *k*_*g*_.

**False discovery rate** To determine the DM status for each CpG site under each cancer type, we calculate the false discovery rates (FDRs) from the parameter estimates. The probability that CpG site *g* is DM in cancer type *c* is calculated as $\text {Pr}\left (H_{gc}=1|\boldsymbol {Z},\hat {\boldsymbol {\Theta }}\right)={\sum \nolimits }_{k=1}^{\hat {K}}\text {Pr}\left (a_{g}=k,H_{gc}=1|\boldsymbol {Z},\hat {\boldsymbol {\Theta }}\right)$. Correspondingly, its local false discovery rate (fdr) [31] is

14 $$ \begin{aligned} \widehat{fdr}_{gc}=\text{Pr}\left(H_{gc}=0|\boldsymbol{Z},\hat{\boldsymbol{\Theta}}\right)=1-\text{Pr}\left(H_{gc}=1|\boldsymbol{Z},\hat{\boldsymbol{\Theta}}\right). \end{aligned}  $$

Following [31] and [58], the global FDR when setting the threshold of local fdr at the cutoff *τ* becomes

15 $$ \widehat{FDR}(\tau)=\frac{{\sum\nolimits}_{g=1}^{G}{\sum\nolimits}_{c=1}^{C}\widehat{fdr}_{gc}I\left(\widehat{fdr}_{gc}\le \tau\right)}{{\sum\nolimits}_{g=1}^{G}{\sum\nolimits}_{c=1}^{C}I\left(\widehat{fdr}_{gc} \le \tau\right)}.   $$

Consequently, after converting all of the $\widehat {fdr}_{gc}$ to $\widehat {fdr}_{gc}$ for each CpG site in each cancer type, we call CpG site *g* as DM in cancer type *c* if $\widehat {FDR}_{gc}\le t$, where *t* is the level at which we control the global FDR.

**TCGA data collection and pre-processing** We collect level 3 Infinium 450K DNA methylation data for 12 cancer types with at least 20 normal samples (see Additional file 1, Table S3) from the Genomic Data Commons Data Portal [59]. We first call DM for each sample type using the InfiniumPurify function from the R package InfiniumPurify with the tumor purity effects adjusted. InfiniumPurify models the methylation levels of normal samples as a normal distribution and subtracts the normal signals from the tumor samples according to their estimated tumor purities using a linear regression model [22].

Among the 396,065 CpG sites in the real data, there are 115 and 204 missing values for BRCA and UCEC, respectively. As PanDM can naturally incorporate missing data into the model and borrow information from the other CpG sites within the same cancer type and the DM status of the same CpG site in the other cancer types, we retain all of the CpG sites with missing values.

## Supplementary information

**Additional file 1** Supplementary material. Figures S1 - S7; Tables S1 - S3. (PDF)

**Additional file 2** Table S4. PanDM cluster membership for the 396,065 CpG sites. (TXT)

**Additional file 3** Table S5A. Enrichment results of functional analysis using the GREAT tool for PanDM cluster 33 (FDRs are smaller than 0.05 for both the binomial and hypergeometric-distribution-based tests). (Excel)

**Additional file 4** Table S5B. Enrichment results of functional analysis using the GREAT tool for the remaining 36 PanDM clusters (FDRs are smaller than 0.05; folds of enrichment are larger than 2 for both the binomial and hypergeometric-distribution-based tests). (Excel)

**Additional file 5** Table S6. Distribution of X-chromosome-located CpG sites in the 37 PanDM clusters. The proportion of X-chromosome-located CpG sites included in each cluster is also given. A hypergeometric test is conducted to investigate the significance of enrichment for the X-chromosome-located CpG sites in each PanDM cluster. The corresponding *p*-values and FDRs are recorded in the last two columns of the table. (Excel)

## Data Availability

Level 3 DNA methylation Infinium 450K array data for the 12 cancer types (see Additional file 1: Table S3) are available from the Genomic Data Commons Data Portal [59]. These data can be downloaded using the gdc-client tool [60]. The proposed model is implemented in R package “PanDM”, which is freely available at http://www.sta.cuhk.edu.hk/YWei/PanDM.html under GNU General Public License, version 2. The authors confirm that there is no patent application pending for the PanDM.

## Abbreviations

DM: differential methylation
WGBS: whole-genome bisulfite sequencing
ICGC: International Cancer Genome Consortium
TCGA: The Cancer Genome Atlas
EM: expectation maximization
BIC: Bayesian information criterion
FDR: false discovery rate
Q-Q: quantile-quantile
GO: gene ontology
BLCA: bladder urothelial carcinoma
BRCA: breast invasive carcinoma
COAD: colon adenocarcinoma
HNSC: head and neck squamous cell carcinoma
KIRC: kidney renal clear cell carcinoma
KIRP: kidney renal papillary cell carcinoma
LIHC: liver hepatocellular carcinoma
LUAD: lung adenocarcinoma
LUSC: lung squamous cell carcinoma
PRAD: prostate adenocarcinoma
THCA: thyroid carcinoma
UCEC: uterine corpus endometrial carcinoma
