# Supplementary material for: Pan-cancer analysis of differential DNA methylation patterns
Source: BMC Med Genomics. 2020 Oct 22;13(Suppl 10):154. doi: 10.1186/s12920-020-00780-3 (PMC7579968; doi:10.1186/s12920-020-00780-3)
Supplement: Supplementary file 1 — Additional file 1 Supplementary material. Figures S1 - S7; Tables S1 - S3. (PDF) [file 12920_2020_780_MOESM1_ESM.pdf]

Supplementary Materials for "Pan-cancer analysis of differential DNA methylation patterns"

Mai Shi[1], Stephen Kwok-Wing Tsui[1,2], Hao Wu[3] and Yingying Wei[4*]

[1] School of Biomedical Sciences, The Chinese University of Hong Kong, Shatin, New Territories, Hong Kong SAR, China

[2] Hong Kong Bioinformatics Centre, The Chinese University of Hong Kong, Shatin, New Territories, Hong Kong SAR, China

[3] Department of Biostatistics and Bioinformatics, Rollins School of Public Health, Emory University, 1518 Clifton Road, Atlanta, Georgia 30322, USA

[4] Department of Statistics, The Chinese University of Hong Kong, Shatin, New Territories, Hong Kong SAR, China

*To whom correspondence should be addressed.

Email: ywei@sta.cuhk.edu.hk (YW)

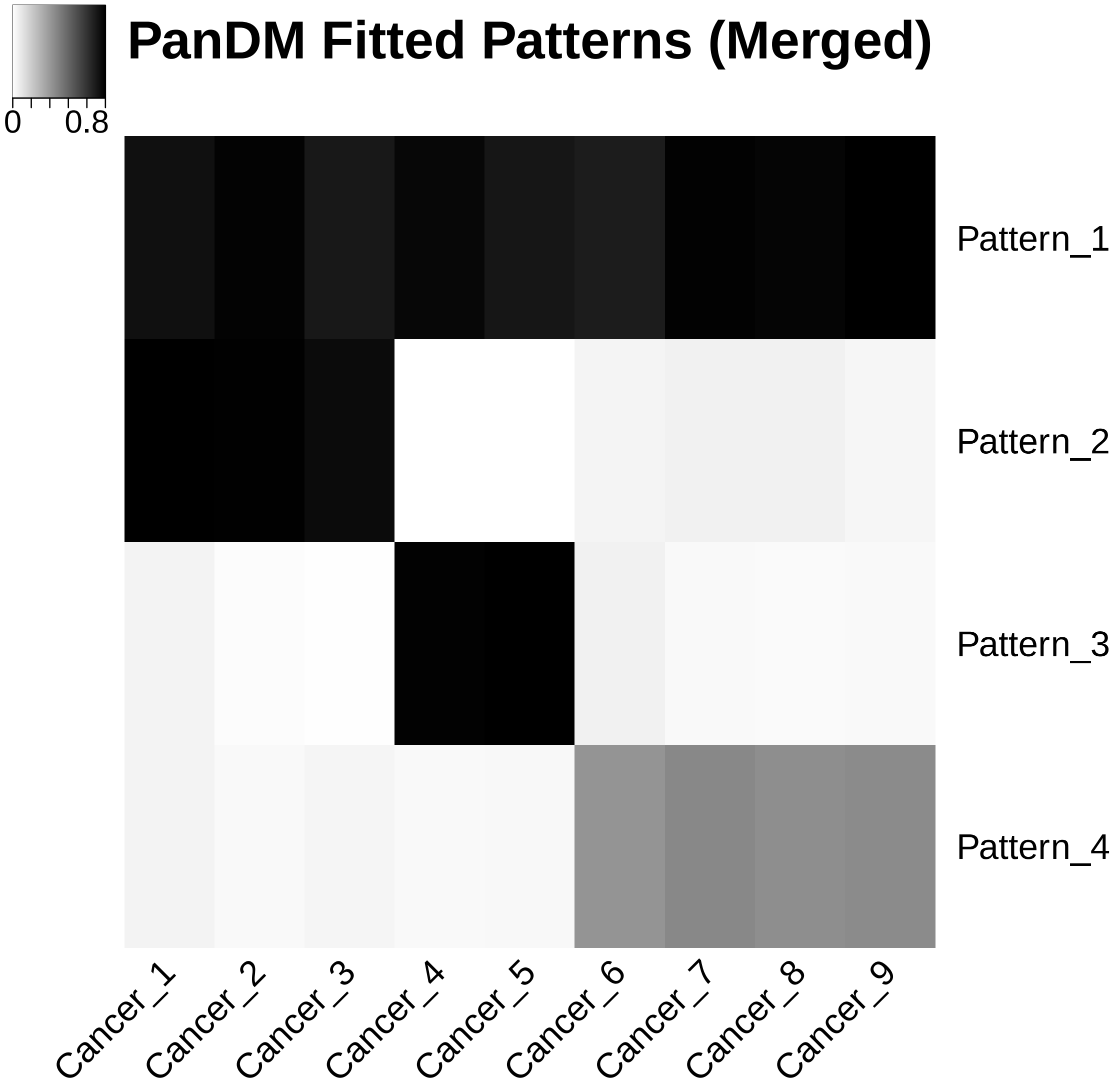

Figure S1: DM patterns learned by PanDM when K = 4. PanDM can still recover the major patterns with two true clusters merged due to a smaller K.

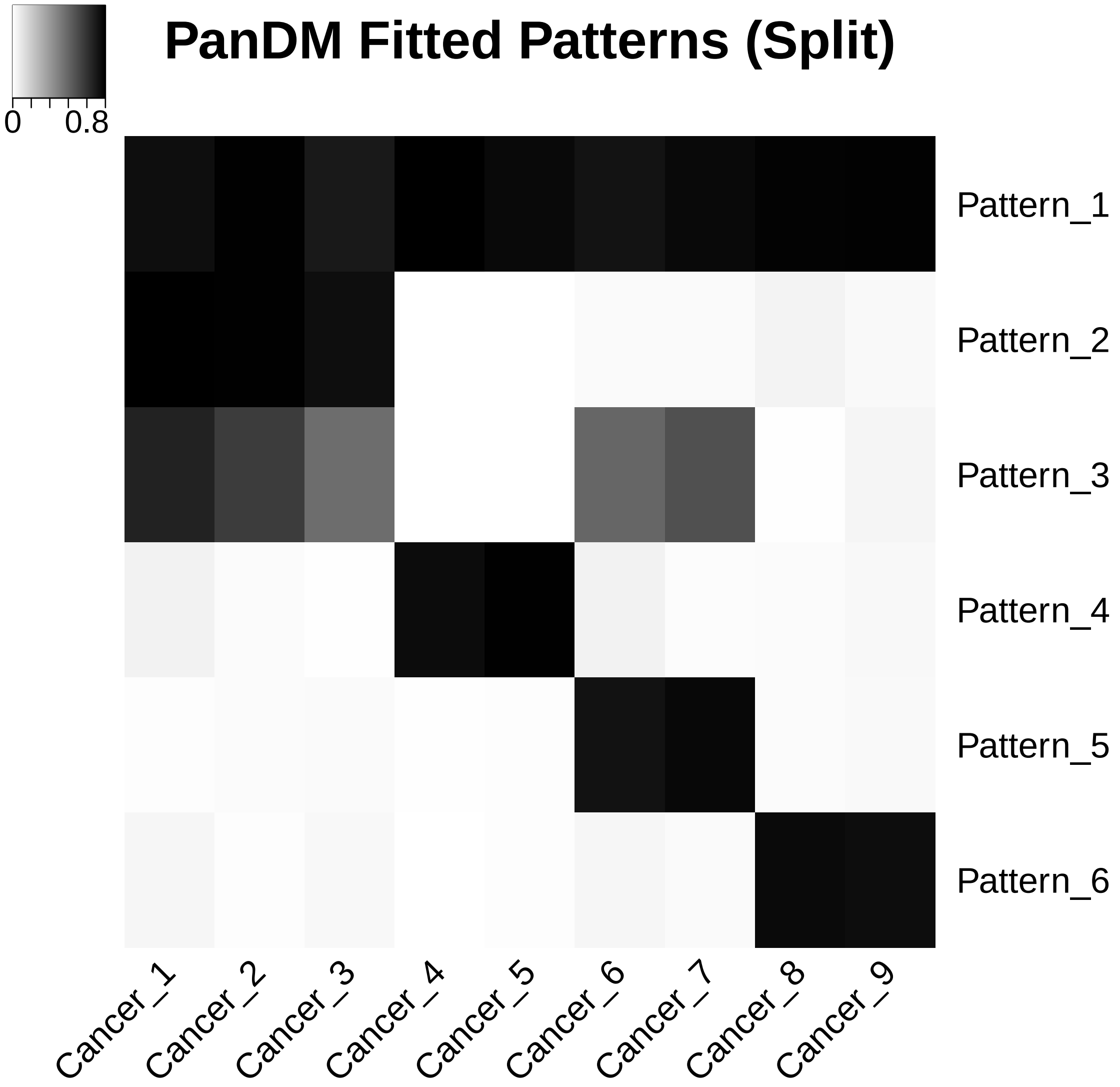

Figure S2: DM patterns learned by PanDM when K = 6. PanDM can still recover the major patterns with one true cluster split into two due to a larger K.

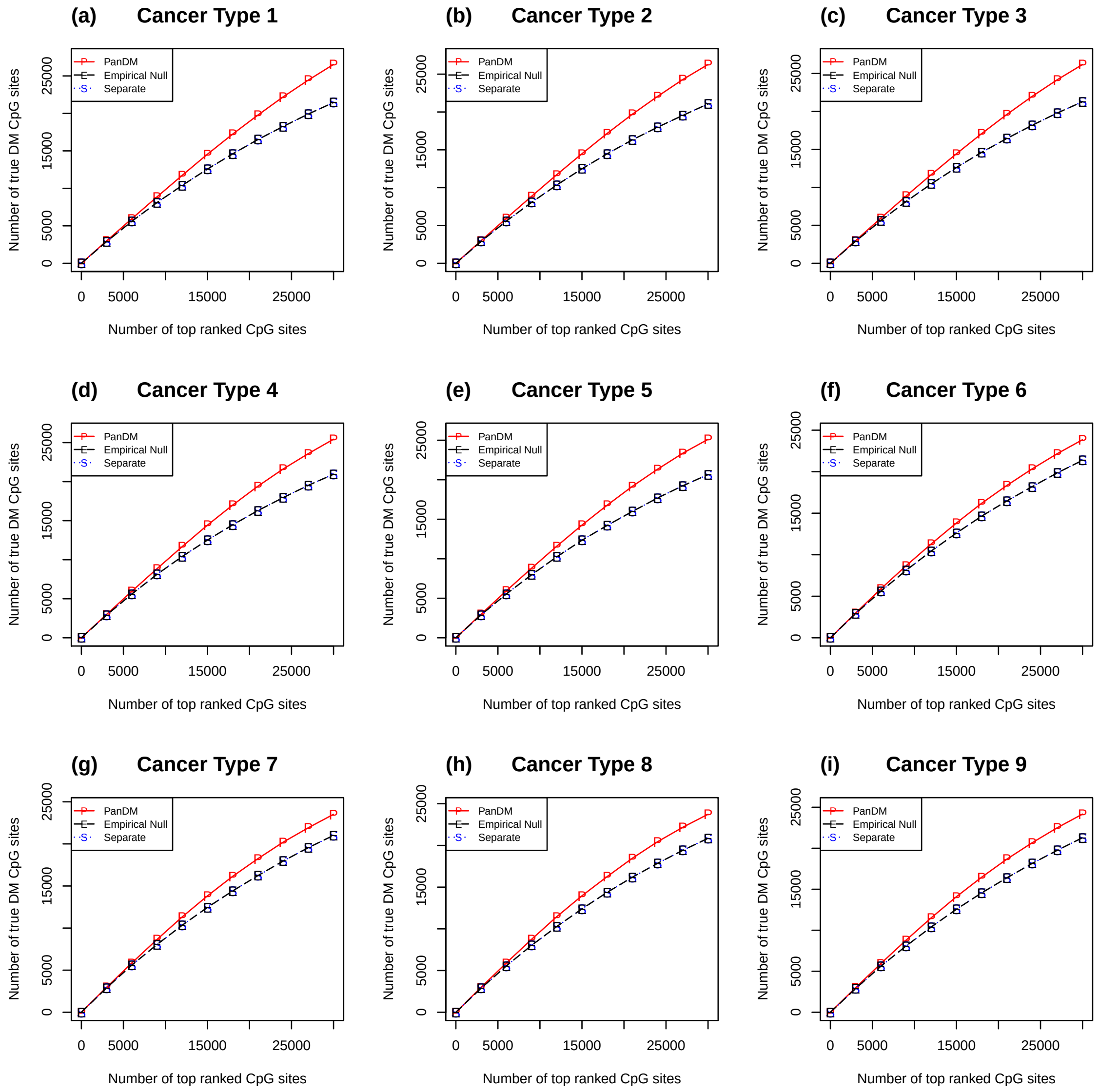

Figure S3: The number of true positives among the top ranked CpG sites by each of the three DM calling methods (K = 4).

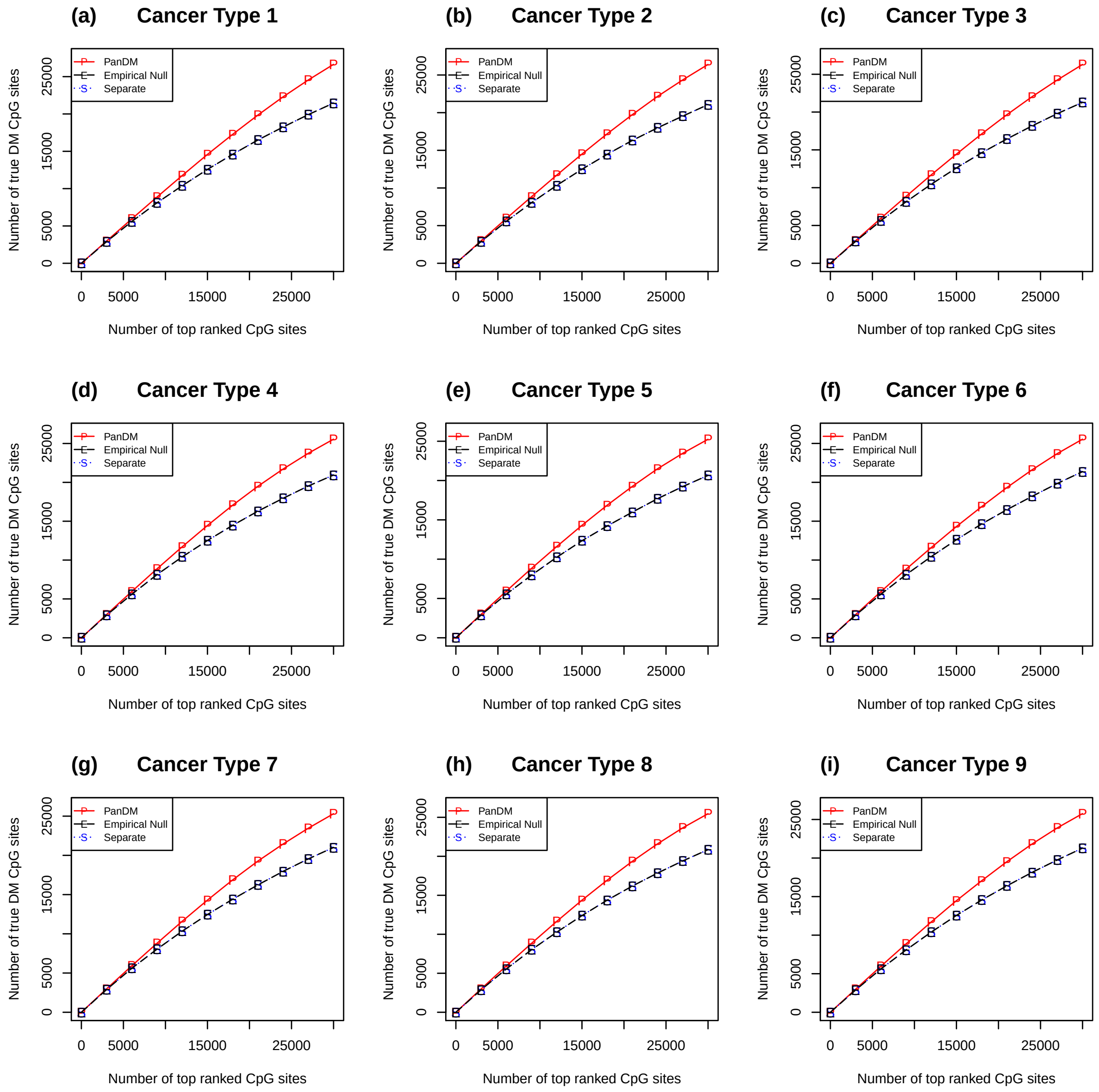

Figure S4: The number of true positives among the top ranked CpG sites by each of the three DM calling methods (K = 6).

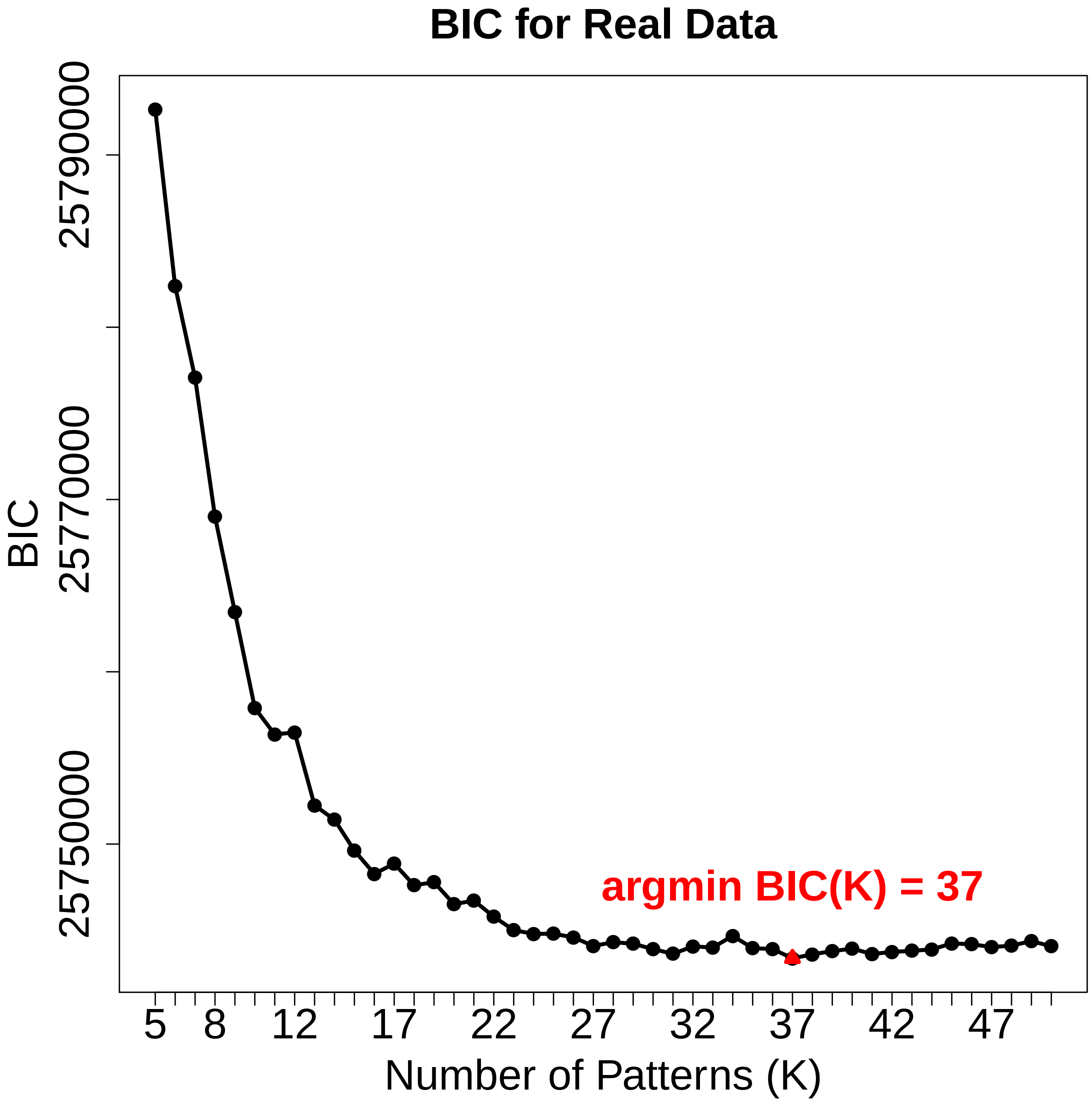

Figure S5: BIC plot for PanDM from $K = 5$ to $K = 50$ on the real TCGA data.The optimal number of patterns is achieved at $K = 37$.

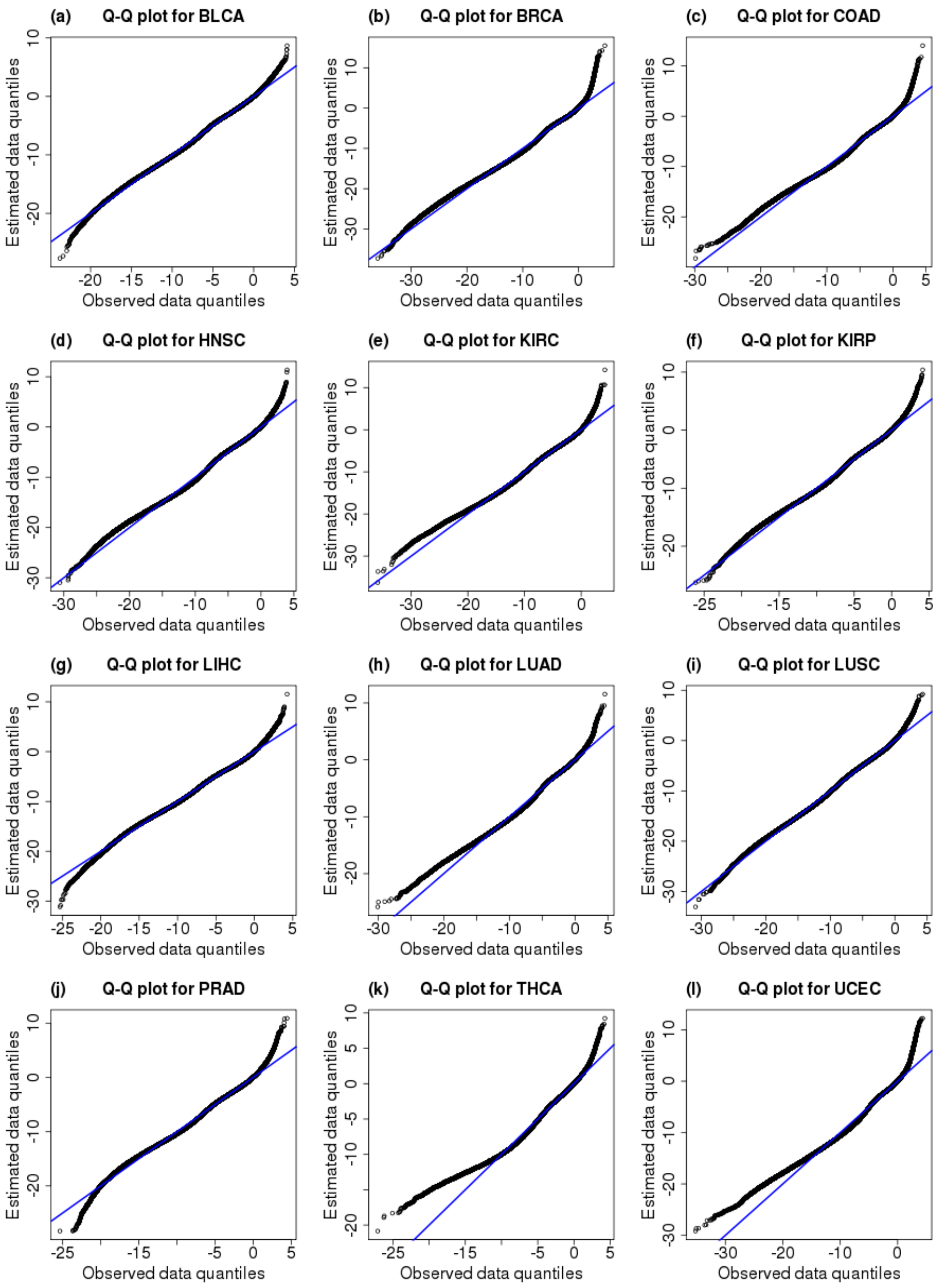

Figure S6: The Q-Q plots for the fitted two-normal mixture versus the real observed tumor-purity-adjusted p-values for the 12 cancer types. The two-normal-mixture distributions estimated by PanDM approximate the marginal distributions of the real data for each cancer type.

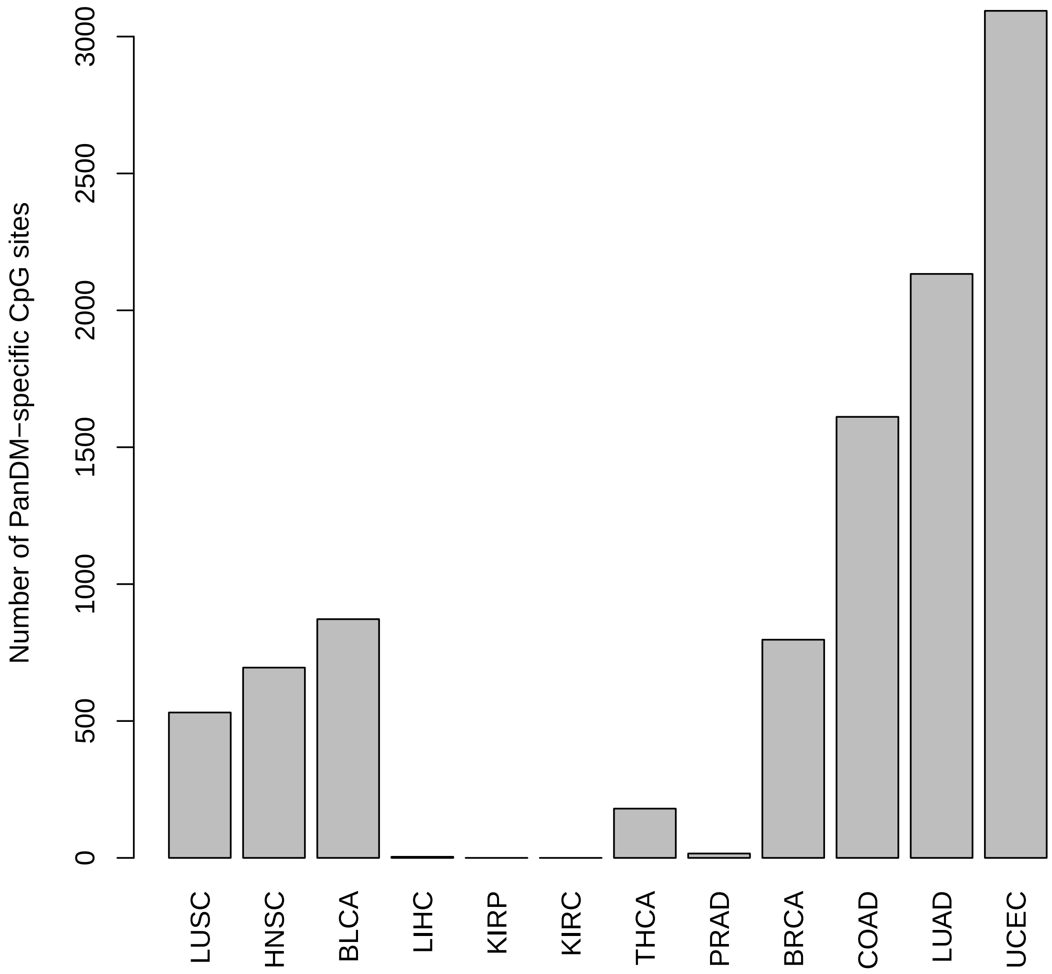

Figure S7: The number of *PanDM-specific CpG sites* identified by PanDM for each cancer type.

	Pattern_1	Pattern_2	Pattern_3	Pattern_4	Pattern_5
Cancer Type 1	0.9640553	0.9937337829	0.037717760	0.032342679	0.036211529
Cancer Type 2	0.9717475	0.9754921899	0.015203595	0.027598088	0.013546449
Cancer Type 3	0.9550829	0.9729307110	0.009290200	0.042754384	0.031283340
Cancer Type 4	0.9885332	0.0027810807	0.980356958	0.008596652	0.009299093
Cancer Type 5	0.9509524	0.0056652081	0.997580770	0.008356333	0.016203526
Cancer Type 6	0.9666804	0.0276856776	0.045967708	0.970397170	0.037388361
Cancer Type 7	0.9623813	0.0259272073	0.017135646	0.966841092	0.006880510
Cancer Type 8	0.9914880	0.0288545207	0.003961512	0.002294950	0.951082681
Cancer Type 9	0.9976509	0.0009530388	0.020211276	0.034982872	0.968326663
π_k	0.1774794	0.2082994	0.1993051	0.2091758	0.2057403

Table S1: The numerals of pattern matrix $(q_{kc})_{K\times C}$ and cluster proportion π_k for the simulation data.

	μ_{c0}	μ_{c1}	σ_{c0}	σ_{c1}
Cancer Type 1	-2.016618	-4.992856	2.503145	2.999790
Cancer Type 2	-1.984987	-4.999668	2.515327	2.986184
Cancer Type 3	-1.995867	-5.013170	2.508411	3.005545
Cancer Type 4	-2.001477	-5.006255	2.482651	3.002497
Cancer Type 5	-2.013811	-4.995807	2.504003	2.998829
Cancer Type 6	-2.010875	-5.000102	2.501666	3.018658
Cancer Type 7	-2.000757	-5.000083	2.501637	2.998386
Cancer Type 8	-2.011726	-4.996300	2.510085	2.992835
Cancer Type 9	-1.985461	-4.990566	2.498068	2.985486

Table S2: The numeral setting of the distributions for the simulation data.

Cancer type	TCGA code	# Tumor samples	# Normal samples
Bladder urothelial carcinoma	BLCA	419	21
Breast invasive carcinoma	BRCA	746	96
Colon adenocarcinoma	COAD	301	38
Head and neck squamous cell carcinoma	HNSC	530	50
Kidney renal clear cell carcinoma	KIRC	325	160
Kidney renal papillary cell carcinoma	KIRP	276	45
Liver hepatocellular carcinoma	LIHC	380	50
Lung adenocarcinoma	LUAD	466	32
Lung squamous cell carcinoma	LUSC	359	42
Prostate adenocarcinoma	PRAD	429	50
Thyroid carcinoma	THCA	515	56
Uterine corpus endometrial carcinoma	UCEC	439	34

Table S3: Overview of the detailed cancer types and number of samples collected from the Genomic Data Commons Data Portal (https://gdc-portal.nci.nih.gov/) for real data analysis.
